# 

**Published:** 1884-09

**Authors:** 


WHOLE NUMBER, CCLXVIII. SEPTEMBER, 1884. NUMBER 2, VOLUME XXIV.

LIBRARY INDEXED S.G.O.

### THE BUFFALO Medical and Surgical Journal.

EDITORS:

THOS. LOTHROP, M. D., A. R. DAVIDSON, M. D.,

PUBLISHED BY THE MEDICAL JOURNAL ASSOCIATION.

No. 8 WEST CHIPPEWA ST.

Entered at the Post-Office at Buffalo, N. Y., as Second-class Mail Matter.

